# Structure of the BRK domain of the SWI/SNF chromatin remodeling complex subunit BRG1 reveals a potential role in protein–protein interactions

**DOI:** 10.1002/pro.3820

**Published:** 2020-01-13

**Authors:** Mark D. Allen, Mark Bycroft, Giovanna Zinzalla

**Affiliations:** ^1^ MRC Laboratory of Molecular Biology Cambridge UK; ^2^ Microbiology, Tumor and Cell Biology (MTC) Department Karolinska Institutet Stockholm Sweden; ^3^ Present address: Petmedix, Granta Park Cambridge UK

**Keywords:** BAF complexes, cancer, heart diseases, neurodevelopmental disorders, NMR, protein–protein interactions, SMARCA4 subunit, structural determination

## Abstract

BRG1/SMARCA4 and its paralog BRM/SMARCA2 are the ATPase subunits of human SWI/SNF chromatin remodeling complexes. These multisubunit assemblies can act as either tumor suppressors or drivers of cancer, and inhibiting both BRG1 and BRM, is emerging as an effective therapeutic strategy in diverse cancers. BRG1 and BRM contain a BRK domain. The function of this domain is unknown, but it is often found in proteins involved in transcription and developmental signaling in higher eukaryotes, in particular in proteins that remodel chromatin. We report the NMR structure of the BRG1 BRK domain. It shows similarity to the glycine‐tyrosine‐phenylalanine (GYF) domain, an established protein–protein interaction module. Computational peptide‐binding‐site analysis of the BRK domain identifies a binding site that coincides with a highly conserved groove on the surface of the protein. This sets the scene for experiments to elucidate the role of this domain, and evaluate the potential of targeting it for cancer therapy.

## 1 INTRODUCTION

Human SWItch/Sucrose Non‐Fermentable (SWI/SNF) complexes (BRG1/BRM associated factor [BAF] complexes) are ATP‐dependent chromatin remodelers that control gene expression by repositioning nucleosomes.^1^ They are involved in essential cellular processes, such as transcriptional regulation, DNA replication, repair, and recombination. These large complexes are highly dynamic and are assembled combinatorially from multiple subunits (2–5) encoded by more than 29 genes.

Subunits of these complexes are mutated in approximately 20% of human cancers^6^, ^7^, ^8^ with mutations occurring in several different complex subunits. For example, BRG1, a catalytic ATPase subunit, is mutated in numerous cancer types,^9^ including lung cancer, medulloblastoma, and pancreatic cancer.

Conversely, overexpression of specific subunits without mutation is emerging as an alternative mechanism by which cellular transformation can occur. For example, several tumor types present elevated levels of BRG1, and multiple studies have shown that targeting BRG1 suppresses cell proliferation.^9^, ^10^

We have also started to understand that at different stages of certain tumors SWI/SNF complexes can act either as tumor suppressors or as oncogenes.^11^, ^12^, ^13^ These mechanistic insights are providing new therapeutic opportunities, and targeting SWI/SNF subunits has great potential for the development of novel cancer therapies,^2^, ^3^, ^4^, ^5^, ^14^, ^15^ and several efforts are ongoing to develop BRG1 inhibitors.^9^

Human exome sequencing and genome‐wide association studies have revealed that mutations in SWI/SNF‐complex subunits are also linked to several neurodevelopmental disorders.^16^ Targeting SWI/SNF complexes offers moreover great potential for heart diseases^17^ as they play a critical role in cardiac development, congenital heart disease, cardiac hypertrophy, and vascular endothelial cell survival.

**Figure 1:**
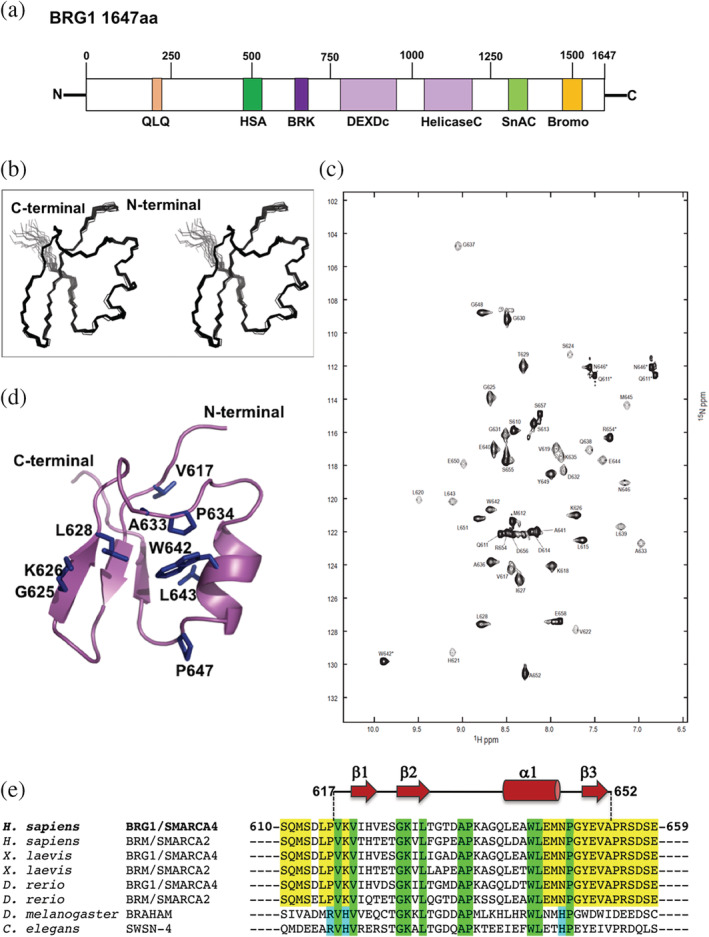
Structure of human BRG1/SMARCA4 BRK domain. (a) Representation of the domain structure of human BRG1/SMARCA4. Numbers across the top of the schematic represent residue number. The ATPase module consists of DEXDc and HelicaseC domains. (b) Stereo view of the overlay of the 20 lowest energy NMR structures of BRG1 BRK (D614‐S655). (c) HSQC spectra of ^3^C/^15^N‐labeled sample. (d) Cartoon representation of the NMR structure of the BRG1/SMARCA4 BRK domain (PDB ID = 6SY2) with the conserved residues indicated. (e) Sequence alignments of BRK domains of SWI/SNF ATPase subunits from *Homo sapiens* (human), *Xenopus laevis* (African frog), *Danio rerio* (zebrafish), *Drosophila melanogaster* (fruit fly), and *Caenorhabditis elegans* (roundworm). Highlighted in green sequence conservation throughout all the species; highlighted in yellow sequence conservation in species that have two different copies of the ATPase subunit (BRG1/SMARCA4 and BRM/SMARCA2); highlighted in cyan sequence conservation in species in which there is only one copy of the ATPase subunit. BRK, Brahma and Kismet domain; Bromo, bromodomain; HSA, N‐terminal helicase‐SANT domain; QLQ, Glutamine‐Leucine‐Glutamine; SnAC, SNF2 ATP‐coupling domain

The mutually exclusive ATPase subunits BRG1/SMARCA4 and BRM/SMARCA2 are core components of SWI/SNF complexes. They are multidomain proteins that contain both DNA and protein interaction modules (Figure 1a). These paralogs share 86% similarity in their amino acid sequence. In addition to the ATPase module, which is a member of the SNF2 family of ATPases, they also have a bromodomain that interacts with acetylated lysines on histone H3 and H4 tails, and an HSA domain, which binds to nuclear actin. Both BRG1 and BRM also possess a BRK domain located between the HSA domain and the ATPase module. The BRK domain is a 40 amino acid sequence motif also found in a second family of chromatin remodeling enzymes that includes the protein Kismet in fruit fly, and the chromodomain helicases CHD7, CHD8, and CHD9 in mammals.^18^ These proteins have one or two copies of the BRK domain, which in contrast to BRM and BRG1 are located C‐terminal to the ATPase module. The domain is also found in the Drosophila protein (PPS) that regulates alternative splicing.^19^ The function of the BRK domain is unknown. BRG1 and BRM have been implicated in a wide range of protein–protein interactions (BioGRID interaction database^20^), but none of these have been mapped specifically to the BRK domain. As part of a program in our laboratory to establish the role of and to target domains of the SWI/SNF complex,^21^, ^22^ we set out to determine the structure of the human BRG1 BRK domain.

## 2 RESULTS AND DISCUSSION

Examining the sequence of BRG1/SMARCA4 and BRM/SMARCA2 in a range of species, we identified a highly conserved region between residues 610 and 659, encompassing the BRK domain that is flanked by less conserved regions that are predicted to be intrinsically disordered (InterPro database). We expressed and purified this fragment, obtained the NMR assignments of ^3^C/^15^N‐labeled protein using conventional heteronuclear methods,^23^ and determined its solution structure by NMR (Figure 1b–d, Table 1). This revealed a structured module between residues V617 and A652 (Figure 1d), with two proline residues (P616 and P653) forming the boundaries of the folded region. The BRK domain has a beta‐beta‐alpha‐beta fold (Figure 1d), with the first beta strand forming the middle strand of an antiparallel beta sheet.

**Table 1:** Summary of conformational constraints and statistics for the 20 accepted NMR structures of the human BRG1/SMARCA4 BRK domain

Structural constraints	
Intraresidue	352
Sequential	232
Medium range (2 ≤ |i‐j| ≤ 4)	97
Long range (|i‐j| > 4)	205
Dihedral angle constraints (*side chain*)	8
TALOS constraints	78
Distance constraints for 12 hydrogen bonds	24
Total	988
Statistics for accepted structures	
Statistical parameters (± *SD*)	
Rms deviation for distance constraints	0.0046 Å ± 0.0006 Å
Rms deviation for dihedral constraints	0.127 ° ± 0.018 °
Mean CNS energy term (kcal Mol^−1^ ± *SD*)	
E (overall)	29.62 ± 0.88
E (van der Waals)	5.53 ± 0.55
E (distance constraints)	1.48 ± 0.34
E (dihedral and TALOS constraints)	0.17 ± 0.05
Rms deviations from the ideal geometry (*SD*)	
Bond lengths	0.0009 Å ± 0.00004 Å
Bond angles	0.319 ° ± 0.0023 °
Improper angles	0.125 ° ± 0.006 °
Average atomic rmsd from the mean structure (*SD*)	
Residues 184–249 (N, Cα, C atoms)	0.340 Å ± 0.077 Å
Residues 184–249 (all heavy atoms)	0.741 Å ± 0.085 Å
Structural quality	
Residues in most favored region of Ramachandran Plot	98.0 ± 2.0%
Residues in additional allowed region of Ramachandran Plot	2.0 ± 2.0%
Residues in disallowed region of Ramachandran Plot	0.0 ± 0.0%

**Figure 2:**
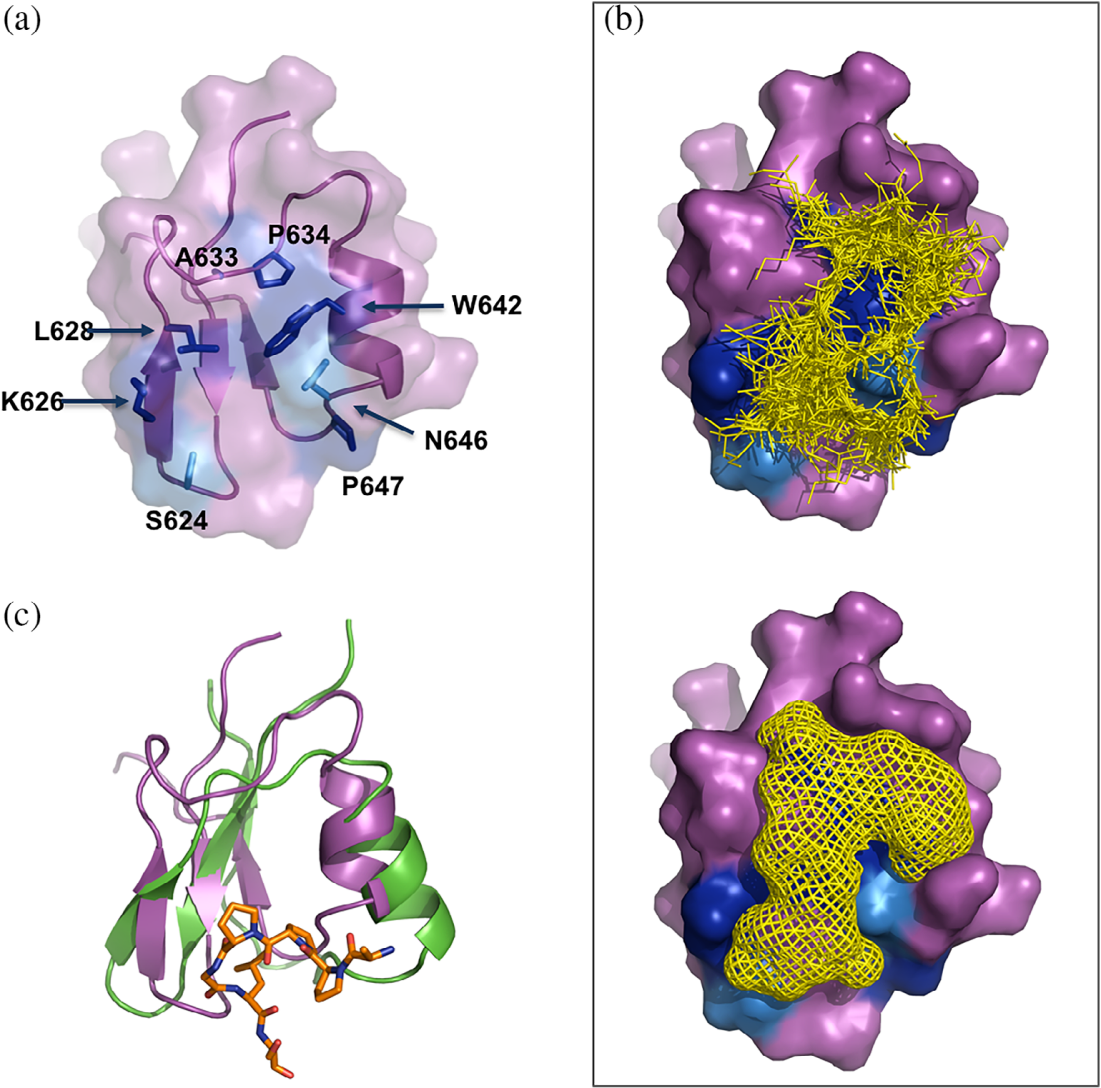
Predicted peptide binding site on the BRK domain. (a) Cartoon and surface representation of the BRK domain structure showing the conserved surface residues: strictly conserved ones in dark blue and in light blue the serine residue 624 and the asparagine 646. (b) Cartoon representation of the output of the peptide binding site prediction by ACCLUSTER (yellow lines, top) and by PeptiMap (yellow mesh, bottom) on the BRK domain (surface representation). (c) Overlay (cartoon representation) of the NMR structure of the BRK domain (in magenta) and the crystal structure of the GYF domain of Smy2 (in green) in complex with a proline‐rich peptide from BBP/ScSF1 highlighted in orange (PDB = 3FMA)

Alignments of the region used for structure determination for BRG1 and BRM proteins from vertebrates as well as homologues from fly and worm, which only contain one SWI/SNF ATPase subunit is shown in Figure 1e. Several amino acids are conserved throughout all the species, including lower metazoans possessing only one copy of the ATPase subunit. All of these strictly conserved residues are found within the structured region (Figure 1e): V617, V619, and L643 form part of the hydrophobic core and are conserved to maintain the structure of the fold. The other residues are all solvent exposed and cluster on one face of the domain in a shallow groove (Figure 2a). G625, K626, and L628 are in beta 2 that forms one side of the groove. The top of the groove is formed by the loop between beta 2 and the helix 1 that contains A633 and P634. The other side of the groove is formed by one face of the helix that contains W642. The bottom of the pocket is formed by the loop connecting the helix with beta 3 that contains another strictly conserved proline P647. Residue N646 (Figure 2a) located on the face of the helix that forms part of the shallow groove is conserved in BRG1 and BRM proteins and undergoes a conservative substitution to a histidine residue in species that have only one copy of the ATPase subunit. Residue S624 that is also situated with in the conserved surface grove (Figure 2a) remains as a serine in all the other BRG1 homologs, but is substituted with a threonine in BRM proteins and in the BRK domains of the ATPase subunits from lower metazoans. The surface residues on the opposite face of the domain are not conserved.

The residues flanking the structured region are conserved from zebrafish to human in both BRG1 and BRM. These residues likely constitute short‐linear‐interaction motifs (SLIMs) either mediating intra‐ or inter‐molecular protein–protein interactions. These sequences appeared at the same time as the duplication of the SWI/SNF ATPase subunit into the BRG1 and BRM paralogs, and may reflect a more‐complex regulation of SWI/SNF complexes in higher vertebrates. Interestingly two serine residues 610 and 613 in the N‐terminal flanking region have been reported to be sites for phosphorylation in multiple studies (PhosphoSitePLus database^24^).

We thought that the groove that contains the conserved surface residues (Figure 2a) could be a site for peptide binding. We thus ran two widely used computational programs for the detection of peptide binding sites. The first one, PeptiMap^25^ takes into account peptide binding site characteristics, the second, ACCLUSTER,^26^ uses the 20 standard amino acids as probes to globally scan the surface of a given protein. PeptiMap represents the most probable binding site on the protein as a mesh, while ACCLUSTER presents the identified peptide binding site as a cluster of amino acid fragments depicted as lines (Figure 2b). Both programs give strikingly similar results predicting the shallow groove as the primary site for peptide binding (Figure 2b).

Structures of the two BRK domains from CHD7^27^ and one of the two BRK domains of CHD8 (PDB ID = 2CKA) have been determined. All these structures have the beta‐beta‐alpha‐beta fold present in the BRG1 structure. The second CHD7 BRK domain has two additional C‐terminal helices that pack onto the face of the domain opposite to the putative binding site identified in the BRG1 BRK domain. The CHD8 BRK domain has one additional C‐terminal helix that also packs onto this face of the domain. The residues corresponding to the putative binding site are highly conserved within domains from CHD7 and related proteins, which suggests that this region is also functionally important in this family of proteins.^27^ Some of the residues within this site are conserved in both families of proteins while others are conserved only within one group of proteins suggesting that there may be functional differences between these two families of BRK domains. In the SCOP database^28^ the BRK domain is grouped together in a fold with the GYF domain.^29^ The GYF domain is a well‐characterized peptide recognition domain, which binds to its target using a binding site located in a similar position to the one identified in the BRK domain (Figure 2c). There are some differences between the two domains, in particular the orientation of the helix. The GYF domain binds a short proline‐rich short peptide motif (Figure 2c). The difference in orientation of the helix in the BRK domain opens up the surface groove that appears to allow it to bind a longer peptide motif.

Taking all of this in consideration this study strongly suggests that the BRK domain acts as a peptide‐recognition module within BRG1 and its paralog BRM. It may mediate interactions between subunits or, alternatively, maybe involved in binding of transcription factors that have been reported to directly interact with this subunit of the SWI/SNF chromatin remodeling complex.

The structure of the BRK opens up a number of avenues to explore the function of this module. The highly context‐dependent roles of SWI/SNF complexes makes chemical biology approaches a particularly attractive strategy for this endeavor. The use of peptidomimetic chemical probes,^30^, ^31^, ^32^ for example, would help to elucidate the role of the domain and assess the potential of targeting it for therapeutic intervention. With regard to druggability with small molecules, an analysis of the structure using the program GHECOM^33^ identifies two pockets for small‐molecule binding within the putative peptide binding site: one shallow pocket adjacent to the conserved AP motif in loop between the second beta strand and the helix and a deeper pocket around the highly conserved tryptophan residue in the helix (W642). Proteolysis targeting chimera (PROTAC) molecules have been used to induce the degradation of several SWI/SNF subunits.^34^ To date, bromodomains have been used as targets for these PROTACs. The BRK domain could be useful as an alternative “handle” for PROTAC molecules to target BRG1 or BRM for degradation as the more limited distribution of this domain compared to bromodomains, could give access to reagents with greater specificity.

Our structural studies will be a useful tool to help to elucidate the complex network of interactions that regulate the functions of SWI/SNF complexes.

## 3 METHODS

### 3.1 *Protein expression and purification*

The DNA encoding residues 610‐659 of human SMARCA4 were amplified from human cDNA by PCR and cloned into a modified pRSETA (Invitrogen) expression vector that produces proteins fused to N‐terminally His_6_‐tagged lipoyl‐domain of *Bacillus stearothermophilus* dihydrolipoamide acetyltransferase. The resulting plasmid was transformed into *Escherichia coli* C41 (DE3) cells. Cells were grown in 2XTY media at 37°C to mid‐log phase and induced with 1 mM IPTG. The temperature was reduced to 22°C and the cells were grown for a further 16 hr. Isotopically labelled BRK domain was prepared by growing cells in K‐MOPS minimal media containing ^5^NH_4_Cl and/or [^13^C]‐glucose. Cells where lysed by sonication, and the fusion protein was purified by Ni^2+^‐NTA affinity chromatography and then dialyzed overnight over night at 4°C in in the presence of TEV protease, which cleaves the BRK domain from the lipoyl‐domain. A second Ni^2+^‐NTA affinity chromatography step was carried out to remove the lipoyl domain and the BRK domain was further purified by size‐exclusion chromatography (SEC).

### 3.2 *NMR spectroscopy*

All spectra were acquired using either a Bruker DRX800 or DRX500 spectrometers equipped with pulsed field gradient triple resonance at 20°C, and referenced relative to external sodium 2,2‐dimethyl‐2‐silapentane‐5‐sulfonate (DSS) for proton and carbon signals, or liquid ammonia for that of nitrogen. Assignments were obtained using standard NMR methods using ^13^C/^15^N‐labeled, ^15^N‐labeled, 10%^13^C‐labeled, and nonlabeled BRG1 BRK samples.^35^, ^36^ Backbone assignments were obtained using the following standard set of 2D and 3D heteronuclear spectra: ^1^H‐^15^N HSQC, HNCACB, CBCA(CO)NH, HNCACO, HNCO, HBHACONH, and ^1^H‐^13^C HSQC. Additional assignments were made using 2D TOCSY and DQF‐COSY spectra. A set of distance constraints were derived from 2D ^1^H‐ and 3D ^1^H‐^15^N NOESY spectra recorded from a 1.5 mM sample with a mixing time of 100 ms. Assignment were made using ANSIG 3.3.^37^ Proton assignments were 96% complete.

### 3.3 *Structure determination*

Distance restraints were obtained from the analysis of 2D 1H and 3D‐15N‐ NOESY spectra integrated according to the cross‐peak strengths and calibrated by comparison with NOE connectivities obtained for standard inter‐residue distances within an α‐helices. After calibration, the NOE constraints were classified into the following categories: strong, medium, weak, and very weak, corresponding to interproton distance constraints of 1.8–2.8, 1.8–3.5, 1.8–4.75, and 2.5–6.0 Å, respectively. Hydrogen bond constraints were included for a number of backbone amide protons whose signals were still detected after 10 min in a 2D ^1^H‐^15^N‐HSQC spectrum recorded in D_2_O at 278 K (pH 5.0). Candidates for the acceptors were identified using the program HBPLUS for the hydrogen bond donors that were identified by the H–D exchange experiments. When two or more candidates of acceptors were found for the same donor in different structures, the most frequently occurring candidate was selected. For hydrogen bond partners, two distance constraints were used where the distance ^(D)^H‐O^(A)^ corresponded to 1.5–2.5 Å and ^(D)^N‐O^(A)^ to 2.5–3.5 Å. Torsional angle constraints were obtained from an analysis of C′, N, C_α_ H_α_, and C_β_ chemical shifts using the program TALOS+.^38^ The stereospecific assignments of H_β_ resonances and chi‐1 angles were determined from DQF‐COSY and HNHB spectra and were confirmed by analyzing the initial ensemble of structures. Stereospecific assignments of H_γ_ and H_δ_ resonances of Val and Leu residues, respectively, were assigned using a fractionally ^3^C–labelled protein sample.^39^ The three‐dimensional structures of the BRK domain was calculated using the standard torsion angle dynamics‐simulated annealing protocol in the program CNS 1.2.^40^ Structures were accepted where no distance violation was greater than 0.25 Å and no dihedral angle violations >5°. Figures were made with the program PyMOL (Schrödinger, LLC). The assignments and the NMR restraints have been deposited in Biological Magnetic Resonance Bank (BMRB) with the ID code 34437.

### 3.4 *Protein data bank accession number*

The atomic coordinates have been deposited in the Protein Data Bank, ID 6SY2.

## CONFLICT OF INTEREST

The authors declare no competing financial interests.

## AUTHOR CONTRIBUTIONS

G.Z. and M.B. designed this study. M.B. and M.D.A. performed the experiments. G.Z. and M.B. analyzed the data, drafted the main manuscript, and prepared all of the figures. All authors reviewed the manuscript.
